# From IL-23/IL-17 to GM-CSF: how is the immuno-inflammatory continuum reshaped in spondyloarthritis?

**DOI:** 10.3389/fimmu.2026.1786912

**Published:** 2026-09-17

**Authors:** Junliang Jia, Wei Xie, Jiawang Zhou, Weidong Wu, Shuliang Zhou

**Affiliations:** Department of Orthopedics, Suzhou Ninth People’s Hospital, Soochow University, Suzhou, Jiangsu, China

**Keywords:** biomarkers, endotypes, entheseal inflammation, GM-CSF, IL-17 ecosystem, IL-23/IL-17 axis, myeloid amplification, osteoproliferation

## Abstract

Spondyloarthritis (SpA) encompasses axial SpA/ankylosing spondylitis, psoriatic arthritis, reactive arthritis, and inflammatory bowel disease-associated arthritis, unified by entheseal inflammation, extra-musculoskeletal manifestations, and a strong but atypical genetic signature. Although SpA lacks the highly specific autoantibodies and immune-complex pathology typical of prototypic autoimmune rheumatic diseases, adaptive immune participation is evident, positioning SpA along an immuno-inflammatory continuum bridging autoimmunity and autoinflammation. The IL-23/IL-17 axis has provided a powerful framework linking barrier perturbation and tissue stress to neutrophil-rich inflammation and to therapeutic efficacy of cytokine blockade. However, IL-17 is increasingly best viewed as an “ecosystem” output generated by multiple lymphocyte lineages, including innate-like cells, under tissue-specific constraints, with partial uncoupling from continuous IL-23 dependence in selected sites and disease stages. These features expose the limits of a linear IL-23/IL-17 model and help explain therapeutic heterogeneity and the imperfect coupling between inflammation control and osteoproliferative outcomes. In parallel, granulocyte-macrophage colony-stimulating factor (GM-CSF) is emerging as a myeloid amplifier that links lymphocyte activation to durable monocyte and macrophage effector programs, reinforcing cytokine redundancy, including TNF and IL-1 family circuits, and promoting inflammatory “lock-in”. We propose a “stage × tissue” model in which early IL-23/IL-17 licensing transitions toward a GM-CSF-licensed, IL-17-dominant, partially IL-23-uncoupled, myeloid lock-in phase. We also discuss biomarker strategies integrating cytokine-module readouts with imaging to support precision trials.

## 1 Introduction

Spondyloarthritis (SpA) comprises a spectrum of interrelated conditions defined by shared clinical, imaging, and classification features (1, 2). Axial SpA/ankylosing spondylitis, psoriatic arthritis, reactive arthritis, and inflammatory bowel disease-associated arthritis share inflammation at the enthesis and adjacent bone and frequent extra-musculoskeletal manifestations (uveitis, psoriasis, and gut inflammation), together with a strong but atypical genetic imprint, especially in axial SpA/AS, where HLA-B27 and antigen-processing pathways are central (3, 4). Unlike prototypic autoimmune rheumatic diseases, SpA lacks highly specific autoantibodies and classic immune-complex pathology, yet it is not a purely autoinflammatory disorder: T-cell-skewed signatures, antigen-presentation genetics, and tissue-resident lymphocyte populations argue for meaningful adaptive immune participation (5). SpA can be viewed as a “bridge” disease within the immuno-inflammatory continuum, where tissue stress and barrier disruption first activate innate immune pathways and are later reinforced by lymphocyte-mediated responses (5, 6). In the past decade, the IL-23/IL-17 axis has provided a useful framework for explaining this biology. It links barrier and entheseal inflammation with neutrophil-rich immune activation, and also helps account for the clinical efficacy of cytokine-targeted therapies in selected SpA settings (7, 8). However, increasing evidence indicates that IL-17 is not generated through a single linear IL-23-dependent pathway. Instead, it can be produced by multiple innate-like and tissue-resident lymphocyte populations, and in certain tissues or disease stages its production may become partially uncoupled from continuous IL-23 signaling (8, 9). In parallel, granulocyte-macrophage colony-stimulating factor (GM-CSF) has emerged as an important amplifier of myeloid effector circuits, connecting lymphocyte activation with chronic synovio-entheseal inflammation and systemic inflammatory burden (10, 11). In this framework, we use IL-23/IL-17 “licensing” to describe the process by which local tissue cues and cytokine signals confer competence for IL-17 effector programs, particularly at the enthesis, without necessarily requiring systemic adaptive immune activation; as used here, licensing denotes a reversible, acquired priming that can wax or wane as local signals fluctuate, rather than a fixed genetic alteration. Biomechanical and barrier-derived cues at the gut and enthesis may therefore promote IL-17-centred effector programs, whereas downstream GM-CSF, together with TNF and IL-1 family cytokines, may drive inflammatory “lock-in”. Here, “lock-in” refers to a self-sustaining, myeloid-dominant effector state maintained by cytokine redundancy and reinforcing feed-forward loops, in which multiple parallel circuits sustain inflammation independently of any single upstream driver and coexist with aberrant tissue repair; in this usage it reflects a functionally stabilized, largely epigenetically reinforced state rather than a heritable genetic change (12, 13).

## 2 The IL-23/IL-17 axis: contributions, constraints, and why it is no longer sufficient

The IL-23/IL-17 axis has become a dominant scaffold for interpreting SpA because it links immunobiology to several defining clinical and tissue features, including neutrophil-rich inflammation, barrier-associated comorbidities, and the demonstrable efficacy of cytokine blockade across diverse therapeutic domains (7, 8). In the canonical view, IL-23, produced by activated dendritic cells and macrophages in response to microbial signals and tissue stress, licenses and stabilizes IL-17-competent programs, classically through Th17 differentiation and maintenance, a scheme that has been refined as the field has moved beyond a purely Th17-centred picture (14, 15). IL-17A and IL-17F then act on tissue-resident mesenchymal and epithelial cells to induce chemokines and antimicrobial peptides, and to amplify secondary inflammatory mediators that recruit and activate myeloid effector cells (16, 17). In synovio-entheseal tissues, this cascade provides a coherent mechanism for turning relatively modest initiating inputs into robust inflammatory recruitment, while also offering a plausible explanation for the frequent coupling of joint disease with the immune perturbations linked to psoriasis and the gut (8, 17). Clinically, inhibition of IL-17 signaling can yield substantial improvements in articular disease and skin inflammation. Secukinumab and ixekizumab have demonstrated efficacy in axial SpA, and dual IL-17A/IL-17F blockade by bimekizumab extends this effect across radiographic and non-radiographic axial SpA (7, 18, 19). Secukinumab is also effective in psoriatic arthritis (20). These clinical data reinforce the view that IL-17 can behave as a convergent effector “output” for diverse upstream triggers (7).

However, the same therapeutic and mechanistic experience that validated this axis has clarified why it cannot be treated as a sufficient standalone explanation of SpA pathogenesis. First, the assumption that IL-23 always acts upstream of IL-17 is not a universal rule. Within structures adjacent to the enthesis in particular, IL-17 can be sustained independently of continuous IL-23 signaling. Cuthbert et al. demonstrated that tissue-resident human entheseal γδT cells produce IL-17A without detectable IL-23R transcripts, indicating that local entheseal tissue-resident programmes and cytokine cues can support IL-17 output independently of detectable IL-23R expression (9). This finding helps explain why IL-23 blockade underperforms in axial disease when IL-17 arises from sources that do not require IL-23, as illustrated by the failure of ustekinumab and risankizumab in axial SpA (21, 22).

A linear model in which IL-23 simply sits upstream of IL-17 also fails to explain how SpA inflammation evolves from fluctuating, anatomically restricted lesions into more entrenched, systemic, and myeloid-dominant states. At this stage, acute-phase responses become more evident, and cytokine redundancy becomes a practical problem rather than a theoretical network concept (12). Feedback loops involving TNF and IL-1β family mediators may continue to sustain inflammation even after one upstream pathway has been blocked (12, 23). This distinction highlights an important clinical problem: control of inflammation does not necessarily mean control of structural progression. In SpA, symptomatic improvement and reduced local inflammatory activity do not always prevent aberrant new bone formation, suggesting that tissue-remodeling pathways partly intersect with, but also diverge from, classical upstream IL-23/IL-17 signaling (24, 25).

For this reason, the IL-23/IL-17 axis should not be treated as a linear, self-contained pathogenic cascade. It is better placed within a broader inflammatory network shaped by anatomical site, mechanical stress, and local immune composition. Two points are especially important. First, IL-17 production is not restricted to a single IL-23-dependent pathway. Its cellular sources and regulatory cues vary across the enthesis, synovium, and intestinal mucosa, reflecting the tissue-specific nature of SpA inflammation (9, 26). Second, models of SpA pathogenesis need to account for downstream amplification loops that can maintain tissue inflammation after early IL-23/IL-17 “licensing” becomes less dominant. Myeloid-centered circuits are particularly relevant here, because they can convert lymphocyte-derived cytokine signals into persistent inflammatory activity and, in some patients, contribute to myeloid “lock-in” (12, 23). These circuits are likely shaped by non-classical IL-17-producing cells and by microenvironment-specific recruitment of neutrophils, monocytes, and macrophages.

## 3 The IL-17 ecosystem in SpA: multiple cellular sources and tissue-specific deployment

The IL-17 axis in SpA is better viewed as a tissue-shaped inflammatory program than as a simple Th17-cell pathway. Across the enthesis, synovium, and gut-associated immune compartments, IL-17 can be produced by several lymphocyte populations, but the relevant source varies with local activation thresholds and tissue constraints (9, 27, 28). Th17 cells remain important, yet rapid IL-17 release may also come from innate-like or unconventional T-cell populations (26). γδ T cells are particularly relevant at barrier and entheseal sites because they can respond to innate cytokine cues with limited dependence on sustained antigen-specific priming. At the human enthesis, γδ T cells can produce IL-17A even when IL-23R transcripts are not detectable, supporting the view that local IL-17 production is not always strictly IL-23-dependent (9, 29). MAIT cells provide a mucosal route into the same pathway; in AS, IL-7 can prime MAIT cells for IL-17 production, linking microbial-metabolite sensing to a type 17 cytokine program (30). Other compartments add further sources: IL-17/IL-22-producing ILC3 populations are expanded in AS, while IL-17A^+^ Tc17 cells have been described in psoriatic arthritis synovium (27, 28, 31). The central issue, therefore, is not simply which cell type produces IL-17, but where the cytokine is released and which inflammatory setting it helps to build (16, 17) (**Table 1**).

**Table 1:** The IL-17 ecosystem in SpA: cellular sources, tissue niches, and network consequences.

IL-17 source/niche	Typical triggers & upstream dependence	Functional consequences in SpA	Key interpretation
Th17 cells	Classically IL-23-licensed/maintained; adaptive activation context (14, 15).	Sustains IL-17 effector programs; amplifies stromal/epithelial activation and myeloid recruitment (16, 17).	Important contributor but not exclusive driver of IL-17 output (no single linear cascade) (8, 15).
γδ T cells	Innate cytokines and stress signals; rapid response; potentially reduced need for sustained antigen priming (9, 29).	Early/rapid IL-17 bursts at barrier or stressed tissues; supports local neutrophil/monocyte recruitment (9, 16, 17).	Supports IL-17 production that can be partly IL-23-independent in certain contexts (9).
MAIT cells	Microbial-derived metabolites; mucosal enrichment; links dysbiosis/barrier perturbation to IL-17 output (30).	Connects gut perturbation to peripheral inflammation; contributes IL-17 in SpA-relevant settings (30).	Mechanistic bridge between microbiome signals and joint/entheseal inflammation (30, 36).
iNKT cells & ILC3	Innate-like activation; can produce IL-17/IL-22-family programs; adds redundancy (27, 29).	Adds non-conventional sources of IL-17/IL-22 in SpA-relevant tissues (27, 29).	Redundancy helps explain why IL-17 output can persist beyond a single Th17-centred pathway (27, 29).
Neutrophil-rich milieu (indirect)	Feed-forward chemokine loops; inflammatory microenvironment amplifies IL-17 responsiveness (16, 17, 23).	Enhances tissue sensitivity to IL-17; stabilizes recruitment loops and propagation (16, 17, 23).	IL-17 effects scale via myeloid recruitment and local amplification, not only source frequency (16, 17, 23).
Enthesis niche	Mechanical stress and tissue perturbation; entheseal resident and innate-like immune programs can support local IL-17-centred inflammation (9, 32, 33).	Favors tissue-driven initiation; IL-17 may arise from resident entheseal T-cell populations and mechanically stressed tissue niches (9, 33).	Consistent with an autoimmunity-autoinflammation continuum and site-specific, partly IL-23-independent IL-17 production (9, 32).
Synovium (peripheral SpA/PsA)	Inflammatory lymphocyte-rich environment; stromal-immune interactions are prominent (28, 31).	IL-17A+ CD8+ cells can co-produce TNF and GM-CSF; distinctive CD8+ T-cell subpopulations are present in SpA synovium (28, 31).	Supports transition toward chronic, myeloid-amplified inflammation (28, 31).
Gut compartment	Context-dependent mucosal IL-17 biology; dysbiosis and IL-17 blockade risk are relevant in SpA (35, 37).	Explains comorbidity patterns and trade-offs of IL-17 pathway inhibition (35, 37).	The same cytokine pathway can have different consequences in mucosa versus joints and skin (35, 37).
Network scaling to chronicity	IL-17 induces chemokines recruiting neutrophils/monocytes; myeloid accumulation adds TNF/IL-1 loops (16, 17, 23).	Feed-forward loops blur upstream/downstream distinctions; cytokine redundancy emerges (12, 23).	Sets stage for GM-CSF as a bridge to durable myeloid effector states (11, 13, 23).

This point is clearest at the enthesis. Rather than being a passive tendon- or ligament-to-bone insertion site, the enthesis is a mechanically loaded fibrocartilaginous interface. Experimental arthritis models show that mechanical strain can influence where inflammation and tissue damage arise (32, 33). In this niche, resident or rapidly recruited innate-like lymphocytes may respond to local stress before a classical systemic adaptive response is fully established, helping place enthesitis within the autoimmunity–autoinflammation continuum (5, 6). The synovium represents a different setting. In peripheral SpA and psoriatic arthritis, available data point to expanded inflammatory lymphocyte programs, including pathogenic CD8^+^ T-cell populations that may reinforce chronic joint inflammation (28, 31). The gut is more difficult to interpret. Subclinical intestinal inflammation and dysbiosis connect mucosal immunity to joint disease through the gut–joint axis (34–36), but IL-17 also contributes to epithelial barrier defense. This dual role is clinically evident in Crohn’s disease, where IL-17A blockade failed and was associated with more adverse events, despite the efficacy of IL-17 inhibition in joint and skin disease (37).

The distribution of IL-17 across these compartments helps explain why SpA inflammation does not always remain confined to the initial site of tissue stress. By inducing chemokines that recruit neutrophils and inflammatory monocytes, IL-17 can gradually turn a local lesion into a more myeloid-rich inflammatory environment (16, 17). In this sense, tissue-specific IL-17 production may mark the transition from IL-23/IL-17 “licensing” toward downstream myeloid amplification and, in some patients, myeloid “lock-in.” As myeloid cells accumulate, they can provide additional TNF and IL-1 family mediators, increasing tissue responsiveness to IL-17 and sustaining inflammation through a feed-forward circuit that makes the distinction between “upstream” and “downstream” cytokines less clear in established disease (12, 23). In this context, the relative proportions of IL-17 sources that rely on IL-23 and IL-17 sources that do not rely on IL-23 become key factors determining treatment responsiveness; At the same time, downstream factors that maintain the survival and activation of myeloid cells have gradually become candidate amplification mechanisms. As a result, GM-CSF has received increasing attention as a link between lymphocyte activation and inflammatory arthritis, including SpA and persistent myeloid effector status (11, 13).

## 4 GM-CSF and the myeloid amplification loop: a bridge from lymphocyte licensing to chronic effector states

If IL-17 is the versatile effector output in SpA, GM-CSF functions as a key amplifier, converting upstream immune activation into sustained myeloid-dominant inflammation. GM-CSF thus bridges adaptive and innate-like lymphocyte responses to the effector machinery of monocytes, macrophages, dendritic cells, and granulocytes (13). GM-CSF promotes the survival, differentiation, and inflammatory programming of myeloid cells, increasing both the magnitude and the duration of tissue inflammation (38). As emphasized by Wicks and Roberts, GM-CSF plays a distinctive, non-redundant part in this process, regulating the production, recruitment, and activation of myeloid cells in ways not fully duplicated by other proinflammatory mediators such as TNF or IL-1β (39). In a network view of SpA, GM-CSF is therefore well positioned to explain how early programmes centered on IL-17 can evolve into disease states marked by cytokine redundancy, high acute-phase activity, and clinical entrenchment, states that are less easily mapped onto a single upstream node (12, 13) (**Figure 1**).

**Figure 1:**
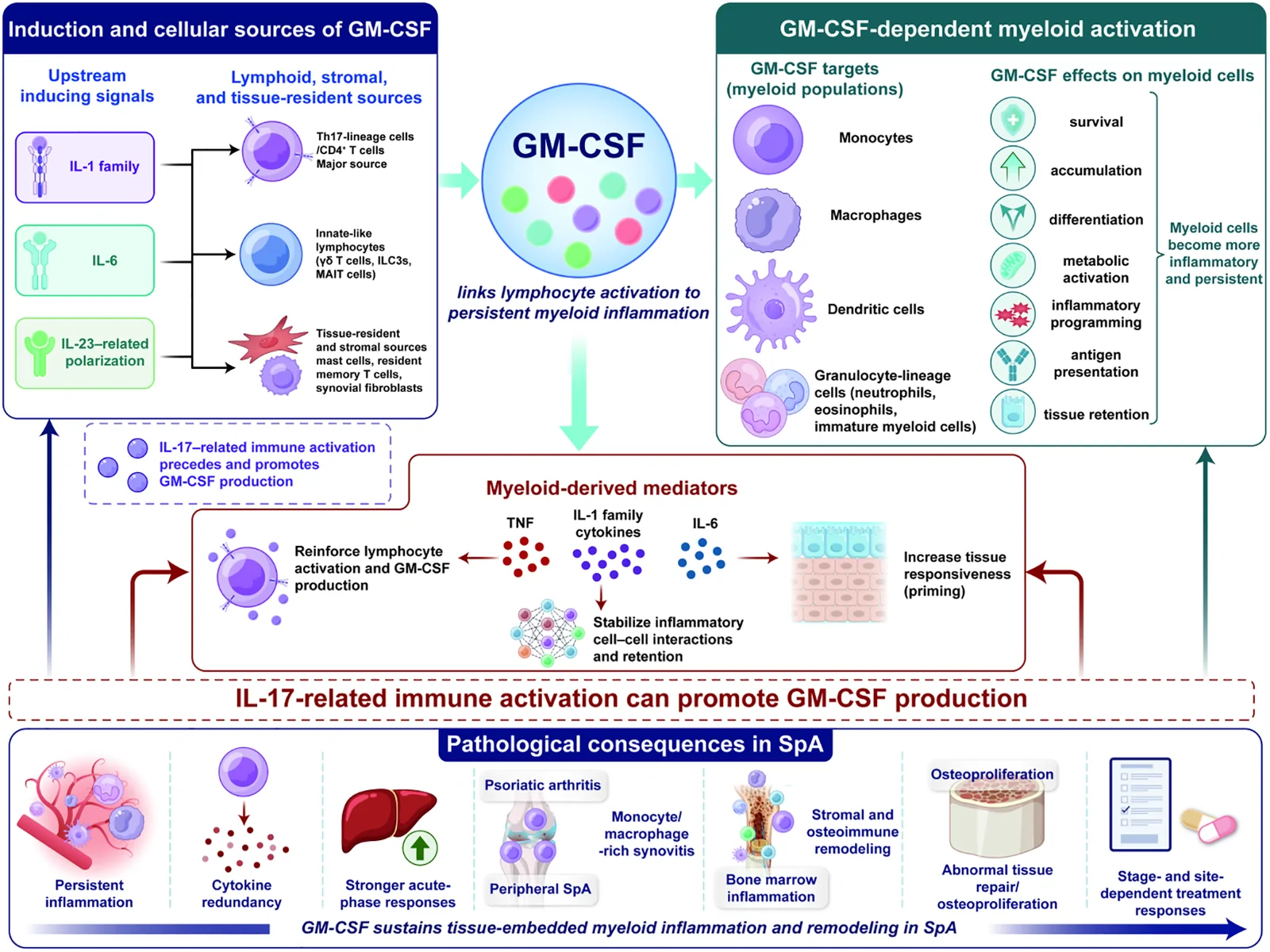
GM-CSF-driven immune amplification and tissue remodeling in spondyloarthritis.

### 4.1 Sources and induction of GM-CSF in SpA-relevant inflammation

GM-CSF production in SpA-relevant inflammation is not confined to a single lymphocyte compartment. Under chronic inflammatory conditions, Th17-lineage cells can acquire the ability to produce GM-CSF. Consistent with this, blood and synovial samples from patients with SpA contain a distinct population of IL-17A^+^GM-CSF^+^ lymphocytes with a specific transcriptional program (11). This overlap is important because type 17 lymphocyte responses do not only provide IL-17-mediated effector activity; they can also deliver GM-CSF signals that support myeloid-cell survival and activation. Nor is GM-CSF restricted to conventional CD4+ T cells. Innate-like and tissue-resident lymphocytes may generate GM-CSF in niches where inflammation is less dependent on classical antigen-driven autoimmunity (11). IL-1 family cytokines, IL-6 and IL-23-associated polarization contexts all sit within this upstream field, making GM-CSF most plausible as a late amplifier that appears as the cytokine network broadens (13, 38). In this staged view, IL-17 first recruits and activates myeloid cells; GM-CSF then increases their persistence and inflammatory fitness.

### 4.2 Mechanisms of myeloid amplification: survival, programming, and feedback

GM-CSF exerts several non-redundant effects on myeloid compartments that make it a plausible driver of chronicity. First, it can enhance the survival and accumulation of monocytes, macrophages, and dendritic cells within inflamed tissues, thereby increasing the cellular substrate available for cytokine production and antigen presentation (13). Second, GM-CSF biases macrophages toward sustained pro-inflammatory states. GM-CSF activated myeloid cells upregulate the production of TNF, IL-1β, and IL-6, establishing a feedback circuit that sustains lymphocyte activation and amplifies local cytokine responsiveness (13, 40). Importantly, this reprogramming may also have an epigenetic component: GM-CSF signals through the JAK2 and STAT5 pathway and can drive transcriptional reprogramming of myeloid cells reminiscent of trained immunity, the innate immune memory in which prior stimulation establishes a relatively long-lived, epigenetically encoded transcriptional state that can amplify subsequent responses (13, 41). Third, GM-CSF can enhance antigen-presenting capacity and tissue retention of myeloid cells, thereby strengthening cellular interactions that sustain chronic inflammation even when the initiating trigger becomes less prominent (13). These effects collectively establish a feed-forward loop: IL-17 and related effector programmes recruit myeloid cells; GM-CSF increases the fitness and inflammatory output of these myeloid populations; the resulting cytokine milieu (TNF, IL-1, IL-6) further maintains lymphocyte activation and tissue sensitivity, closing the circuit (12, 23).

### 4.3 Network integration with TNF, IL-1 family cytokines, and tissue remodeling

GM-CSF amplification gives cytokine redundancy a cellular substrate. Once myeloid cells accumulate, TNF and IL-1 family mediators become both products and reinforcers of the inflammatory circuit; they sustain pro-inflammatory myeloid states, increase IL-17 responsiveness and reduce the likelihood that blockade of any single upstream node will collapse the network (12, 23). In SpA, this logic is supported by elevated serum GM-CSF in axial disease and by its persistence despite TNF inhibitor therapy (10). The implication is not that GM-CSF blockade is already established in SpA, but that persistent GM-CSF activity could help maintain myeloid survival and tissue retention when TNF is suppressed. Anti-GM-CSF receptor strategies therefore remain a testable extension of inflammatory-arthritis therapeutics rather than a validated SpA treatment (42). The same amplifier may also intersect indirectly with tissue repair. SpA couples inflammation to syndesmophyte formation and periosteal new bone, through Wnt, BMP and Dickkopf-1-regulated pathways that are only partly coupled to inflammatory activity (24, 43, 44). Longitudinal imaging sharpens this point: vertebral corner inflammation followed by fat metaplasia predicts some subsequent syndesmophyte growth, yet spinal lesions can continue to drive new bone even under TNF inhibition (25, 45). GM-CSF should therefore be viewed as a bridge from lymphocyte licensing to myeloid dominance, not as a direct structural-progression pathway.

## 5 Crosstalk and therapeutic heterogeneity: integrating stage and tissue to explain response patterns

A network that contains both an IL-17 ecosystem and a GM-CSF-centered myeloid amplifier reframes therapeutic heterogeneity. Divergent patterns of treatment response and structural progression in SpA are unlikely to be mere noise around a single dominant pathway. They may instead reflect a patient’s position across two intersecting dimensions: the maturity of the inflammatory circuit and the tissue context in which that circuit is operating (5, 6). The proposed “stage × tissue” framework is not meant to replace clinical phenotypes. Rather, it adds a mechanistic layer that may help explain several clinical patterns that are otherwise difficult to reconcile. Upstream blockade may fail once downstream effector cytokines remain active; downstream inhibition may still retain efficacy in established disease; and structural progression may remain partly dissociated from inflammatory control (7, 22, 45).

### 5.1 A staged model: from IL-23/IL-17 licensing to myeloid lock-in

In an early IL-23/IL-17 “licensing” phase, local tissue signals are likely to remain important. Barrier perturbation, entheseal stress, and IL-23-associated polarization can promote IL-17 activity before the inflammatory network becomes highly redundant (33, 46, 47). At this point, IL-17 functions as a rapid effector cytokine. It recruits neutrophils and inflammatory monocytes, activates stromal cells, and helps create tissue conditions that permit further immune-cell entry (16, 17). This phase should, in principle, remain sensitive to IL-17 blockade, consistent with the efficacy of IL-17 inhibitors in axial SpA (7, 18, 19) and with supporting evidence from PsA (20).

With repeated recruitment and persistent activation, however, the same circuit may become harder to reverse. Myeloid cellularity increases, acute-phase responses become more evident, and TNF, IL-1 family mediators, and IL-6 begin to sustain inflammation through overlapping feedback pathways (12, 23). GM-CSF becomes particularly relevant at this stage. By supporting myeloid-cell survival and inflammatory programming, it may help maintain inflammatory output even when IL-23/IL-17 “licensing” is no longer the dominant driver (13, 38). Clinically, this shift may help explain persistent systemic inflammation and reduced sensitivity to upstream intervention. It may also explain why IL-17 blockade can retain activity in settings where IL-23 blockade shows less consistent benefit (7, 22). This staged model should not be read as a universal disease clock; rather, it treats inflammatory-circuit maturity as a variable that may shape treatment response and biomarker interpretation (48, 49).

### 5.2 A tissue model: enthesis/axial niches versus synovial/peripheral niches

Stage alone cannot fully explain therapeutic heterogeneity in SpA. The disease has a clear anatomical pattern, with prominent involvement of the enthesis and axial skeleton, although many patients also develop peripheral synovitis or extra-musculoskeletal disease. These compartments differ in baseline immune composition, mechanical load, and stromal organization. Such differences may influence both the dominant cellular sources of IL-17 and the extent to which IL-23/IL-17 “licensing” progresses toward myeloid “lock-in” (50–52).

At the enthesis, mechanical loading and tissue stress engage resident myeloid cells and innate-like lymphoid populations. Human entheseal tissue studies show that local cells can produce IL-23 and IL-17A, at least partly independently of systemic IL-23 signaling (9, 51, 52). Mechanistically, enthesitis lies at the interface between autoinflammatory and autoimmune processes, with local innate responses likely predominating before adaptive circuits become fully established (5). This niche-based biology helps explain why IL-17 blockade can be effective in axial inflammation, whereas upstream targeting has produced less consistent responses across clinical contexts (7, 22).

Peripheral synovium represents a different inflammatory site. In psoriatic arthritis and peripheral SpA, the synovial microenvironment contains expanded inflammatory lymphocyte programs, including IL-17A^+^ CD8^+^ T-cell populations capable of co-producing TNF and GM-CSF (28, 31). In this compartment, clinical response may depend not only on reducing IL-17 output, but also on whether myeloid amplification has progressed toward myeloid “lock-in,” particularly in patients with prominent systemic inflammatory signatures (39).

The gut adds a distinct constraint because IL-17 family cytokines also support epithelial barrier defense. As a result, the balance between protective immunity and pathogenic amplification may differ substantially from that in joints or skin. This helps explain why IL-17 pathway inhibition can have different clinical consequences across SpA manifestations and comorbidities (35, 37). Taken together, these tissue-specific constraints suggest that the same cytokine may occupy different functional positions depending on anatomical site. Therapeutic efficacy and safety therefore cannot be directly extrapolated from one SpA domain to another.

### 5.3 From mechanisms to response patterns: practical implications and testable predictions

Integrating stage and tissue yields several testable predictions that can guide both clinical reasoning and study design. First, patients with signatures of strong myeloid activation, such as prominent acute-phase responses and myeloid-skewed transcriptional profiles, may represent “lock-in” endotypes in which redundant cytokine circuits make upstream interruption less effective and treatment may need to target dominant effector modules rather than a single initiating cytokine (12, 48). Second, disease dominated by axial and entheseal involvement may be enriched for IL-17-driven programmes that can be sustained by innate-like lymphocytes with variable dependence on IL-23, placing IL-17 closer to an essential effector node in that tissue setting (9, 26). Third, disease dominated by peripheral synovitis may more readily engage myeloid amplification supported by GM-CSF, suggesting that pathways related to GM-CSF could be relevant for persistent inflammatory activity and, given the established role of GM-CSF in arthritic pain, potentially for pain in selected patients (11, 53). Finally, because structural progression and osteoproliferation can be partially dissociated from inflammatory control, response assessment may need to combine inflammatory biomarkers with imaging-based measures and, potentially, stromal and osteoimmune readouts that are not captured by cytokine-centric monitoring (25, 45). The final section outlines a provisional endotyping scheme and discusses biomarker strategies that could operationalize “stage × tissue” mechanisms in future trials and precision medicine approaches.

## 6 Translation and outlook: endotypes, biomarkers, and priorities for the next phase of SpA research

Given the complexity of the cytokine network outlined above, clinical phenotypes alone are unlikely to capture the mechanistic states that shape treatment response, relapse, and structural progression in SpA. We therefore propose a provisional endotyping framework aligned with the “stage × tissue” model. One pattern reflects an IL-23/IL-17 “licensing” state, in which IL-17 output remains closely linked to upstream polarization cues. Another reflects an IL-17-dominant, partly IL-23-uncoupled state, particularly in anatomical niches where innate-like lymphocytes sustain IL-17 production in response to local stress or alternative cytokine signals. A third endotype is defined by myeloid “lock-in,” a self-reinforcing state in which cytokine redundancy and persistent monocyte–macrophage programs are stabilized by TNF, IL-1 family mediators, and GM-CSF (11, 48).

Translating these endotypes into research or clinical use will require biomarkers that describe where a patient sits within the inflammatory network, rather than measuring single cytokines in isolation. Useful approaches may include transcriptomic signatures of myeloid and neutrophil activation, circulating or tissue-derived chemokine panels as functional surrogates of IL-17 activity, and composite readouts that link acute-phase reactants with imaging evidence of entheseal stress, synovial inflammation, and structural remodeling (11, 48). The main value of these biomarkers would be to distinguish patients whose disease remains largely dependent on IL-23/IL-17 “licensing” from those who have entered myeloid “lock-in,” where downstream cytokine redundancy may reduce the predictive value of upstream pathway markers.

Several research steps are needed to test this framework. Longitudinal molecular sampling across different anatomical compartments could help define how, and when, early IL-23/IL-17 “licensing” gives way to myeloid “lock-in.” Multi-omic cohorts should link cytokine modules with treatment outcomes across axial, peripheral, and extra-musculoskeletal disease domains. Future trials may be more informative if patients are stratified by mechanistic signatures, including IL-23/IL-17 “licensing,” GM-CSF-linked myeloid activation, and myeloid “lock-in,” rather than by diagnostic category alone. In parallel, inflammation and structural progression should be tracked as related but separable outcomes, because symptomatic improvement does not necessarily mean that osteoproliferation has been halted (25, 49).
